# The Effect of Alcohol on Telomere Length: A Systematic Review of Epidemiological Evidence and a Pilot Study during Pregnancy

**DOI:** 10.3390/ijerph18095038

**Published:** 2021-05-10

**Authors:** Andrea Maugeri, Martina Barchitta, Roberta Magnano San Lio, Maria Clara La Rosa, Claudia La Mastra, Giuliana Favara, Marco Ferlito, Giuliana Giunta, Marco Panella, Antonio Cianci, Antonella Agodi

**Affiliations:** 1 Department of Medical and Surgical Sciences and Advanced Technologies “GF Ingrassia”, University of Catania, 95123 Catania, Italy; andrea.maugeri@unict.it (A.M.); martina.barchitta@unict.it (M.B.); roberta.magnanosanlio@phd.unict.it (R.M.S.L.); mariaclara.larosa@unict.it (M.C.L.R.); claudia.lamastra@unict.it (C.L.M.); giuliana.favara@unict.it (G.F.); marco.ferlito@studium.unict.it (M.F.); 2 Obstetrics and Gynecology Unit, Department of General Surgery and Medical Surgical Specialties, University of Catania, 95123 Catania, Italy; giunta.giuliana@studium.unict.it (G.G.); mpanella@unict.it (M.P.); acianci@unict.it (A.C.)

**Keywords:** alcohol consumption, alcohol abuse, biological aging, telomere length, drinking

## Abstract

Several studies—albeit with still inconclusive and limited findings—began to focus on the effect of drinking alcohol on telomere length (TL). Here, we present results from a systematic review of these epidemiological studies to investigate the potential association between alcohol consumption, alcohol-related disorders, and TL. The analysis of fourteen studies—selected from PubMed, Medline, and Web of Science databases—showed that people with alcohol-related disorders exhibited shorter TL, but also that alcohol consumption per se did not appear to affect TL in the absence of alcohol abuse or dependence. Our work also revealed a lack of studies in the periconceptional period, raising the need for evaluating this potential relationship during pregnancy. To fill this gap, we conducted a pilot study using data and samples form the Mamma & Bambino cohort. We compared five non-smoking but drinking women with ten non-smoking and non-drinking women, matched for maternal age, gestational age at recruitment, pregestational body mass index, and fetal sex. Interestingly, we detected a significant difference when analyzing relative TL of leukocyte DNA of cord blood samples from newborns. In particular, newborns from drinking women exhibited shorter relative TL than those born from non-drinking women (*p* = 0.024). Although these findings appeared promising, further research should be encouraged to test any dose–response relationship, to adjust for the effect of other exposures, and to understand the molecular mechanisms involved.

## 1. Introduction

Telomeres are repeated sequences of TTAGGG forming DNA–protein structures at the end of each chromosome with a function of preventing genomic instability [1]. In recent decades, telomere length (TL) has been proposed as a marker of biological aging because it gradually shortens in somatic cells throughout life, as a result of no expression of telomerase [2]. While telomeres protect the chromosomes from DNA damage and ensure genomic stability, telomere shortening is considered as a marker of the cumulative damage to which cells are progressively subjected [3]. On the other hand, however, telomere shortening is also considered a cause of biological aging, dictating the life span of a cell [3]. Although TL is highly inherited—with heritability estimates ranging from ~30% to ~80% [4]—only a small proportion of the genetic variants in telomere maintenance genes explains this variance [4]. More recently, an epigenome-wide association study of leukocyte TL has also demonstrated that DNA methylation of more than 800 CpG sites is associated with TL, and that these loci are within genes involved in circadian rhythm, coagulation, and wound healing [5]. These findings partially explain why TL considerably varies between individuals, even among those of the same chronological age, and suggest the involvement of other factors, including environmental exposures, behaviors, and diseases [5]. Its presumed role in biological aging, in fact, means that TL has emerged as a potential biomarker of age-related diseases, such as cancer and cardiometabolic diseases [6]. On the other hand, however, there is now also consensus that risk factors (e.g., physical inactivity, smoking cigarettes, unhealthy diet, etc.) might affect TL before the abovementioned diseases develop [4,7].

As reported by the World Health Organization (WHO), alcohol abuse still remains a main risk factor for human health, with more than 3 million deaths and 130 million disability-adjusted life years attributable to excessive alcohol consumption in 2016 [8]. For this reason, some studies investigating the association of TL with age-related diseases included alcohol consumption as a covariate in their analyses [9,10,11]. Similarly, others began to study the extent to which drinking alcohol and other lifestyles were associated with TL in depressive and anxiety disorders [12]. Yet, most of these studies just considered alcohol consumption as a confounder or mediator of the relationship between TL and the disease under investigation. Only more recently have some research groups begun to focus on the primary effect of alcohol consumption and related disorders on TL. However, a clear interpretation of their findings was hindered by differences in study design, characteristics of the study population, methods used for measuring telomere length, and other factors taken into consideration. Moreover, their findings were often inconclusive and/or limited to specific groups of patients. For example, to our knowledge, the research on the effect of alcohol consumption on TL during pregnancy is currently lacking. Yet, it has already been demonstrated how adverse exposures during pregnancy (e.g., maternal stress and smoking) might induce telomere shortening in both cord blood [13,14,15,16] and placenta samples [17]. Beyond that, it has also been recognized that adverse maternal conditions (e.g., gestational diabetes and obesity) are associated with telomere shortening in pregnant women, also leading to a higher risk for cardiovascular disease in mothers and their newborns [18,19]. In this scenario, it is also now known that mothers who drink alcohol during pregnancy—especially in the first trimester—jeopardize their health but also that of their children [20], considerably increasing the risk of miscarriage, premature birth, and low birthweight [21]. Although molecular mechanisms underpinning the harmful effects of alcohol consumption during pregnancy are not so clear, telomere shortening represents a plausibly involved factor. However, no studies have to date investigated the effect of maternal alcohol consumption on TL of newborns.

For all these reasons, we first conducted a systematic review of published epidemiological studies to discuss the potential association between alcohol consumption, alcohol-related disorders, and TL. Moreover, we also presented results from a pilot study to give a preliminary description of the putative relationship between maternal alcohol consumption during pregnancy and TL in cord blood at birth.

## 2. Materials and Methods

### 2.1. Systematic Review

#### 2.1.1. Search Strategy

The systematic review was conducted according to the Preferred Reporting Items for Systematic Reviews and Meta-Analyses (PRISMA) guidelines [22] (please consider the PRISMA checklist available in the Supplementary Materials). The literature search was carried out on PubMed, Medline, and Web of Science databases using the following combination of terms applied to all fields of the text: ((Ethanol) OR (Alcohol*) OR (Drink*)) AND ((Telomere) OR (Telomere length) OR (Telomere Shortening)). The last search was done on 12 February 2021, without restrictions on publication date and language.

#### 2.1.2. Study Selection

Included studies had to meet the following eligibility criteria: (1) epidemiological studies of any design (2) evaluating the association of alcohol consumption and/or alcohol-related disorders (3) with TL. By contrast, narrative and systematic reviews, editorials, studies that did not report data about the association, and those that evaluated alcohol consumption only as a covariate of their main analysis were excluded. The literature search and study selection were performed independently by two investigators (AM and RMSL) and disagreements were resolved by discussion with a third investigator (AA).

#### 2.1.3. Data Extraction

For each study, a standard form was used to extract the following data: first author, year of publication, study design, study population, method for assessing alcohol consumption and/or alcohol-related disorders, sample used for DNA extraction, method for measuring TL, results on the association between alcohol consumption, alcohol-related disorders, and TL, and additional findings if present.

### 2.2. Pilot Study

#### 2.2.1. The Mamma & Bambino Cohort

A pilot study was conducted using data and biological samples from the “Mamma & Bambino” project, an ongoing mother–child cohort evaluating the effect of preconceptional, perinatal and early life environment on mothers’ and children’s health [23,24,25,26,27,28]. From 2015, the “Mamma & Bambino” cohort prospectively has recruited pregnant women undergoing prenatal genetic counseling at the Azienda Ospedaliero Universitaria Policlinico “G. Rodolico—San Marco”, Catania, Italy. All pregnant women at 4–20 gestational weeks are invited to participate in the study, which also includes planned follow-ups at delivery and one and two years from birth. Women with multiple pregnancy, pre-existing autoimmune and/or chronic diseases, pregnancy complications (i.e., preeclampsia, gestational hypertension, and gestational diabetes), intrauterine fetal death, and congenital malformations are excluded. The protocol was approved by the ethics committees of the involved institutions, and the study was in line with the Declaration of Helsinki. All participants were fully informed of the purpose and procedures and signed an informed consent to participate.

#### 2.2.2. Assessment of Alcohol Consumption

At recruitment, data on socioeconomic status and lifestyles are collected by trained epidemiologists through structured questionnaires. Specifically, dietary data are collected using the 95-item semiquantitative Food Frequency Questionnaire referring to the month before recruitment [29,30,31,32,33,34,35], and including questions on the consumption of beer, wine, and spirits. For each item, frequency of consumption per week and portion size are recorded. Regarding alcoholic drinks, standard portions refer to a glass of wine, a bottle of beer, or a shot of spirits, which approximately contain 10 g ethanol [36]. It is worth mentioning that, although it is generally recommended to avoid drinking alcohol during pregnancy, a small proportion of participants reports light-to-moderate alcohol consumption (1–7 standard drinks per week) [36].

#### 2.2.3. Biological Samples

The “Mamma & Bambino” project also aims to uncover the effects of maternal exposures on several biomarkers of health and aging [23,24,25,26]. To do that, biological samples from mothers and newborns are collected at recruitment and after delivery. Specifically, we invite women to donate a blood sample at recruitment, an aliquot of amniotic fluid from those who undergo amniocentesis, and a sample of cord blood whenever possible. In this pilot study, we used samples of maternal blood and cord blood to measure leukocyte TL.

#### 2.2.4. Measurement of Telomere Length

Leukocyte genomic DNA was extracted from 200 μL of maternal and cord blood samples using the DNeasy Blood & Tissue kit (Qiagen, Milan, Italy), as described by the manufacturer’s protocol. DNA purification was automated on the QIAcube instrument (Qiagen, Milan, Italy). Concentration and purity of DNA were assessed by a NanoDrop 1000 spectrometer and by a Qubit 3.0 Fluorometer using a dsDNA HS Assay Kit (Thermo Fisher Scientific, Carlsbad, CA, USA). Relative TL was measured using the Relative Human Telomere Length Quantification Assay Kit (ScienCell Research Laboratories, Carlsbad, CA, USA), as described by the manufacturer’s protocol. The real-time quantitative polymerase chain reactions (qPCRs) were performed on a QuantStudio 7 Flex Real-Time PCR System (Thermo Fisher Scientific, Carlsbad, CA, USA) using two sets of primers. The single-copy reference (SCR) primer set amplifies a 100 bp long region on human chromosome 17, serving as a reference for data normalization. The telomere primer set amplifies telomere sequences. Each reaction was performed in a final volume of 20 μL containing 1 μL of DNA (5 ng/μL), 2 μL of primer solution (telomere or SCR), 10 μL of 2X GoldNStart TaqGreen qPCR master mix (ScienCell Research Laboratories, Carlsbad, CA, USA), and 7 μL of nuclease-free water. The PCR conditions were as follows: initial denaturation at 95 °C for 10 min; 32 cycles of 95 °C for 20 s, 52 °C for 20 s, and 72 °C for 45 s. All reactions were run in duplicate and relative TL was expressed as the average of the telomere/single copy reference (T/S) ratio.

#### 2.2.5. Estimation of Statistical Power

The primary hypothesis of this pilot study was that the relative TL of leukocyte DNA from maternal and cord blood samples differed between drinking and non-drinking women. In contrast, the null hypothesis was that maternal alcohol consumption was not associated with relative TL. However, as expected, the proportion of participants who drank alcohol was very small (i.e., nearly 5%). Among them, we identified 5 non-smoking women who completed pregnancy to term (i.e., at 37 weeks of gestation) and who reported drinking alcohol during the first trimester. Thus, assuming an allocation ratio of 1:2, this pilot study had a statistical power of 80% for detecting a mean difference in relative TL of 1 point (standard deviation, SD = 0.5) with a two-sided significance level of 5%. Therefore, we next matched 10 non-smoking women with term pregnancy, who declared not consuming alcohol during the first trimester of pregnancy. The propensity score method was used to match the two groups for maternal age, gestational age at recruitment, pregestational body mass index, and fetal sex.

#### 2.2.6. Statistical Analyses

Statistical analyses were performed using GraphPad Prism (version 6.0, San Diego, CA, USA). Comparisons between drinking and non-drinking women were performed using the Student’s *t*-test or the chi-squared test according to variable type. Relative TL was tested for normality using the Kolmogorov–Smirnov test, expressed as mean and SD, and compared using the Student’s *t*-test. All statistical tests were two-sided, and *p*-values < 0.05 were considered statistically significant.

## 3. Results

### 3.1. Systematic Review

#### 3.1.1. Selection of Studies

**Figure 1:**
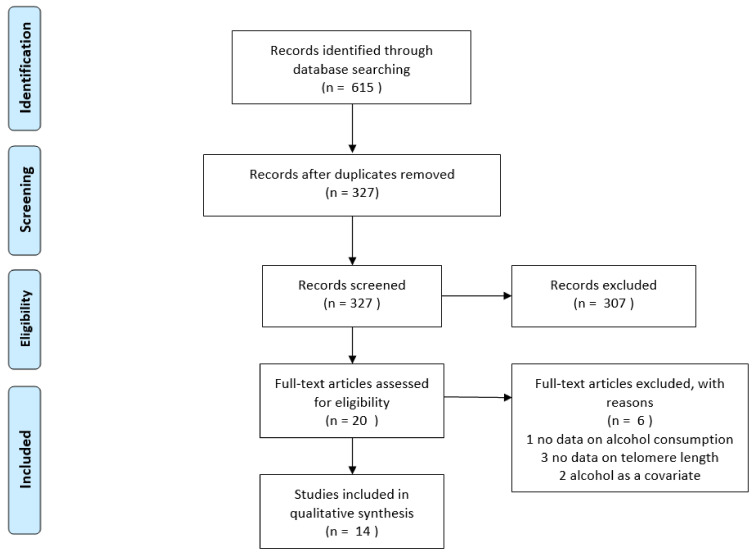
PRISMA flow diagram of study selection.

The study selection process is reported in Figure 1. After removing duplicates, a total of 327 articles were identified from the electronic databases searched. However, 307 were ruled out after reading titles and abstracts and the remaining 20 articles were screened by applying inclusion and exclusion criteria. Among them, one study not reporting information on alcohol consumption, three studies not evaluating the association with TL, and two studies considering alcohol only as a covariate were excluded, while the remaining studies were included in this systematic review.

#### 3.1.2. Study Characteristics

Table 1 summarizes the characteristics of studies included in the present systematic review. Of the 14 studies included, five were conducted in North America, four in Asia, four in Europe, and one in South America. Ten studies featured a cross-sectional design or performed cross-sectional analyses on a prospective cohort, while four studies were prospective. Overall, the sample size ranged from 50 to 5360 participants, who were classified according to alcohol consumption (*n* = 9 studies) or alcohol-related disorders (*n* = 5 studies). Most of the studies (*n* = 12) assessed the TL of DNA extracted from blood samples, while two studies used samples of esophageal mucosa or oral epithelium. With respect to TL assessment, the majority of studies (*n* = 9) used qPCR, while the remaining studies used quantitative fluorescence in situ hybridization (Q-FISH) or Southern blot analysis of terminal restriction fragment lengths.

#### 3.1.3. Telomere Length in Patients with Alcohol-Related Disorders

Studies included in the present systematic review were heterogeneous in terms of objectives, study designs, and results achieved (Table 2). Indeed, some studies compared TL between patients with alcohol use disorders (i.e., compulsive heavy alcohol use and loss of control over alcohol intake) and controls, while others tested the effect of drinking alcohol on TL in other groups of people. The first evidence that alcohol-related disorders might affect TL was published by Aida and colleagues in 2011. The authors compared the TL of DNA extracted from esophageal mucosa of 26 patients with alcohol dependence and that of 24 controls without malignancies in the head and neck, esophagus, stomach, or lung. Interestingly, the normalized telomere-to-centromere ratio appeared to be significantly lower in patients with alcohol dependence than in controls [37]. In the same year, Pavanello and colleagues supported this finding by comparing leukocyte TL between 200 alcohol abusers and 257 controls. Indeed, relative TL was lower in alcohol abusers than in controls, and this difference was independent of their smoking status. The authors also showed that the number of drinks per year was associated with relative TL in the entire population and among alcohol abusers [44]. These findings were further confirmed in recent years by several independent studies. For instance, Yamaki and colleagues assessed leukocyte TL in 48 patients with upper aerodigestive tract cancer and alcohol-related disorders, 86 age-matched alcoholic patients without cancer, and 121 controls without cancer and alcohol-related disorders. In general, their analysis showed shorter TL in patients with alcohol-related disorders if compared with non-alcoholic controls. However, no difference was evident if comparing patients with alcohol-related disorders according to their cancer diagnosis [50]. Similarly, Martins de Carvalho and colleagues demonstrated that alcohol use disorders were associated with lower relative TL. To do that, the authors compared 260 patients with alcohol use disorder and 449 healthy controls. Yet, drinking behaviors were not associated with relative TL in both groups [42]. Finally, the present systematic review identified the study by Tannous and colleagues that compared relative TL in blood samples from 24 patients with alcohol use disorder and 25 controls. Although the difference was not statistically significant, relative TL tended to be shorter in patients with alcohol use disorder [48].

#### 3.1.4. Alcohol Consumption and Telomere Length

In line with the abovementioned evidence, Aida and colleagues also evaluated the association of alcohol consumption with TL. Their study recruited 23 patients with mucosal carcinoma, 12 patients with oral epithelial dysplasia, and 21 controls (i.e., without malignancy in the head and neck, esophagus, stomach, or lung), who were classified as heavy, light, and non-drinkers. Although DNA from oral mucosa of patients with cancer exhibited a lower normalized telomere-to-centromere ratio than healthy controls, no relationship was evident with drinking behaviors in the latter group [38]. In general, most studies also support the absence of a relationship between alcohol consumption and TL in blood samples [39,40,41,43,49]. However, it is worth noting that the prospective study by Strandberg and colleagues demonstrated a cross-sectional association at the baseline but not at the last follow-up [47]. This was partially consistent with the study by Révész and colleagues, which showed a negative association between heavy drinking and relative TL among 2936 participants from the Netherlands Study of Depression and Anxiety [45]. However, the association was evident only at the baseline analysis and it was not significant after adjusting for other covariates [45]. The present systematic review also included a large cross-sectional study by Shin and Baik, who analyzed the potential modifying effects of genetic variants among 1771 participants from the Korean Genome Epidemiology Study. Interestingly, the authors did not find an association between alcohol consumption and relative TL in the overall population. However, they noted an inverse association with heavy drinking among participants with mutant alleles of rs2074356 [46]. This is a single nucleotide polymorphism (SNP) in an uncharacterized locus on chromosome 12, and its mutant allele seemed to be associated with increased risk of esophageal squamous cell cancer.

### 3.2. Pilot Study

**Figure 2:**
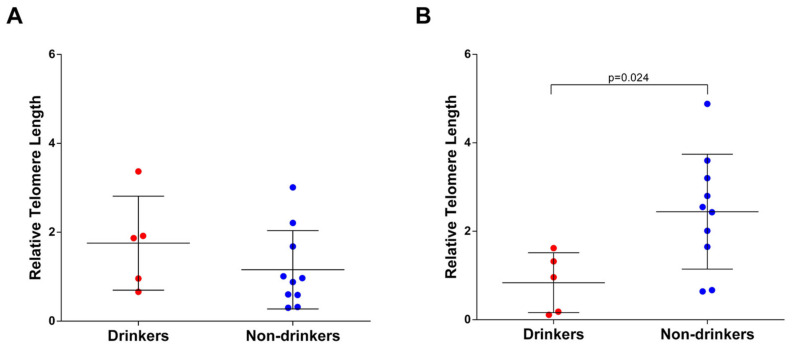
Comparison of relative telomere length between drinkers and non-drinkers in (**A**) maternal blood and (**B**) cord blood.

Due to the lack of evidence on the association between alcohol consumption and TL during pregnancy, here, we report findings of a pilot study conducted on mother–child pairs from the “Mamma & Bambino” cohort. In this cohort, we identified five women who never smoked but who declared habitually drinking alcohol in the first trimester of pregnancy. In addition to the above, we matched 10 non-smoking and non-drinking women, on the basis of maternal age, gestational age at recruitment, pregestational body mass index, and fetal sex (Table 3). The comparison of relative TL measured in leukocyte DNA from maternal blood samples failed to demonstrate any difference between drinkers and non-drinkers (Figure 2A). However, we detected a significant difference when analyzing the relative TL of leukocyte DNA extracted from cord blood samples. Interestingly, newborns from drinking women exhibited shorter relative TL than those born from non-drinking women (Figure 2B).

## 4. Discussion

As demonstrated by Beach and colleagues, both low and high alcohol consumption were associated with accelerated aging assessed by DNA methylation patterns. In contrast, moderate alcohol use was associated with healthy biological aging [51]. Similar to DNA methylation patterns, telomere length can serve as a reasonable proxy for biological aging. However, the evidence on the relationship between alcohol and telomere length is still inconclusive. For this reason, we conducted a systematic review of epidemiological studies on the association of alcohol consumption and alcohol-related disorders with telomere length. Our review revealed two kinds of evidence: the first was that alcohol-related disorders (i.e., diagnosis of alcohol abuse or dependence) were associated with telomere shortening; the second was that alcohol consumption did not appear to affect telomere length in the absence of alcohol-related disorders. This was also confirmed when people were categorized as light, moderate, or heavy drinkers [40], making the possibility of a J-shaped association less likely. However, if the association was non-linear, classifying alcohol consumption at a different threshold could influence the result. Accordingly, future studies should test the shape of the association between alcohol consumption and telomere length, before drawing definitive findings. Previous in vitro research described the effect of moderate concentrations of ethanol on TL dynamics, partially elucidating the mechanism mediating this effect [52]. However, there is still the need for additional experimental studies evaluating physiological and molecular pathways involved in the harmful effect of alcohol on biological aging. Although moderate drinking appears useful for delaying the onset of many age-related conditions (e.g., inflammation, oxidative stress, and apoptosis), there is no robust research on its effect on telomere shortening at the cellular level [53]. In fact, a lot of studies were cross-sectional, while only a few presented well-conducted prospective analyses on sizable cohorts.

Our systematic review also revealed the complete lack of studies during the gestational period. Yet, alcohol abuse has a harmful impact on many targets of the Sustainable Development Goals, including those related to maternal and child health [20]. Drinking alcohol, especially in the first trimester of pregnancy, increases the risk of miscarriage, premature birth, and low birthweight [21]. Due to the lack of evidence, therefore, we designed a pilot study embedded within the “Mamma & Bambino” cohort. Although the study sample consisted of only five drinking and ten non-drinking mothers, our results lay the foundation for deepening the investigation of how maternal alcohol consumption affects biological aging in newborns. On one hand, drinking alcohol in the first trimester of pregnancy did not seem to be associated with the telomere length of leukocyte DNA from the mothers but, on the other hand, it appeared to affect telomere length in DNA from cord blood. Indeed, newborns from drinkers showed shorter telomere length than those born from non-drinkers. This was in line with the developmental origins of health and disease theory that states that in utero exposures program the fetus for challenges that are likely to be experienced later in life [54]. Indeed, our findings—albeit preliminary—support the current hypothesis that biological aging of offspring at birth might reflect maternal and neonatal characteristics related to prenatal environmental adversity [55]. It is worth underlining again that our findings were preliminary and that they did not allow us to draw any conclusion. Yet, they provide further motivations to study telomere shortening as a potential molecular mechanism underpinning the effects of maternal behaviors on the development of chronic disease later in life. However, molecular mechanisms underpinning the effects of maternal alcohol consumption on biological aging of offspring are not yet well understood. Although it is plausible that our findings rely on an alcohol-related state of inflammation and oxidative stress in utero [56], further studies are necessary—if possible, by including more mother–child pairs from different birth cohorts—to shed light onto this relationship.

Our work had some limitations to be considered. Firstly, differences between studies—in terms of study design, study population, assessment of alcohol consumption, and assay used for measuring telomere length—hindered the possibility of applying a meta-analytic approach. In fact, it was not possible to pool studies which assessed and reported alcohol consumption and telomere length in different ways and units of measurement. Secondly, no studies tested the shape of the association of alcohol consumption with telomere length. Moreover, a lot of them, featuring a cross-sectional design, measured telomere length at a single time point. Thirdly, most studies did not adjust their analyses for several factors, such as gender and other lifestyles. Indeed, researchers have to face the possibility that gender disparities might arise from the investigation of behavioral, physiological, and molecular factors. In this scenario, prioritizing the role of gender in this kind of analysis is crucial for a better understanding of the effect of lifestyles on biological aging [57]. Finally, information on alcohol consumption was often collected using ad hoc questionnaires and was therefore prone to inaccuracies that should be controlled with innovative tools for dietary assessment [58]. These weaknesses, along with the limited sample size, were also intrinsic to our pilot study. Our pilot study, in fact, was conducted only on 15 mother–child pairs (i.e., five drinking versus 10 non-drinking mothers). Although we estimated the statistical power of this analysis, we had to consider our results as preliminary and encouraging for further large studies. Moreover, it would be necessary to take into account other maternal behaviors and exposures that might affect telomere length. In our study, for example, we applied a propensity score matching to at least partially manage other factors, including smoking habits, maternal age, gestational age at recruitment, pregestational body mass index, and fetal sex. Accordingly, future studies should be encouraged to consider these and other factors in the analyses. For all these reasons, our results were not meant to be conclusive, but rather we presented them to arouse interest in this emerging field of research.

Given all these considerations, available epidemiological studies showed an inverse association between alcohol-related disorders and telomere length, though drinking alcohol in general did not appear to influence it. Maternal alcohol consumption, instead, might be associated with shorter telomere length in offspring, but the research is only at the preliminary stages. Indeed, further large epidemiological studies should be encouraged to support our findings by testing the shape of any dose–response relationships, by investigating the effect of other exposures, and by uncovering the molecular mechanisms involved.

## Figures and Tables

**Table 1:** Characteristics of studies included in the systematic review.

Study	Study Design	Population	Age (Years)	Gender (% of Men)	Alcohol-Related Classification	Sample	Telomere Length Assessment
Aida et al., 2011 [37]	Cross-sectional	26 alcoholic patients and 24 controls without head and neck, esophagus, stomach, or lung cancer	Mean of 61.2 in alcoholic patients and 73.3 in the control group	100% of alcoholic patients and 50% in the control group	DSM-IV criteria for alcohol dependence	Esophageal mucosa	Quantitative fluorescence in situ hybridization
Aida et al., 2019 [38]	Cross-sectional	21 subjects without head and neck, esophagus, stomach, or lung cancer	Mean of 40.4	57.1%	History of alcohol drinking classified as active drinking and non-active drinking. Active drinkers were also categorized as light drinkers and heavy drinkers	Oral epithelium	Quantitative fluorescence in situ hybridization
Dixit et al., 2019 [39]	Prospective	1675 participants in the Heart and Soul Study and the Cardiovascular Health Study	Mean of 66.8 in the Heart and Soul Study and 74.8 in the Cardiovascular Health Study	81.5% in the Heartand Soul Study and 41.2% in the Cardiovascular Health Study	Alcohol consumption; alcohol type; binge drinking; and ideal drinking	Blood	Southern blot analysis of terminal restriction fragment lengths
Latifovic et al., 2015 [40]	Cross-sectional	477 healthy volunteers	20–50 years	43%	Alcohol consumption categorized into abstainer, low, moderate, and high	Blood	Quantitative real-time PCR
Liu et al., 2013 [41]	Cross-sectional	1715 participants from the Nurses’ Health Study	Median of 59.8	0%	Alcohol intake obtained from Food Frequency Questionnaire	Blood	Quantitative real-time PCR
Martins de Carvalho et al., 2019 [42]	Cross-sectional	260 patients with alcohol use disorder and 449 healthy controls	Mean of 44 in patients with alcohol use disorders and 33.3 in controls	71.9% of patients with alcohol use disorder and 55.2% of controls	DSM-IV criteria for alcohol dependence and drinking behaviors	Blood	Quantitative real-time PCR
Needham et al., 2013 [43]	Cross-sectional	5360 participants from the Nutrition Examination Survey	Mean of 48.6	48%	Alcohol use was classified as heavy and moderate drinking	Blood	Quantitative real-time PCR
Pavanello et al., 2011 [44]	Cross-sectional	200 alcohol abusers and 257 controls	Mean of 38 in alcohol abusers and 44 in controls	100%	Alcohol intake obtained from self-reported questionnaires	Blood	Quantitative real-time PCR
Révész et al., 2016 [45]	Prospective	2936 participants from the Netherlands Study of Depression and Anxiety	18–65	33.6%	Alcohol consumption obtained from questionnaires and categorized into non-drinking, mild–moderate drinking, and heavy drinking	Blood	Quantitative real-time PCR
Shin and Baik, 2016 [46]	Cross-sectional	1771 participants from the Korean Genome Epidemiology Study	49–79	49%	Alcohol consumption obtained from questionnaire-based interviews and categorized into light, moderate, and heavy consumption	Blood	Quantitative real-time PCR
Strandberg et al., 2012 [47]	Prospective	499 men from the Helsinki Businessmen Study	Mean of 47.7	100%	Alcohol consumption obtained from questionnaire-based interviews	Blood	Southern blot analysis of terminal restriction fragment lengths
Tannous et al., 2019 [48]	Cross-sectional	24 patients with alcohol use disorder and 25 controls	Mean of 47.0 in patients with alcohol use disorder and 43.8 in controls	75% of patients with alcohol use disorder and 68% in controls	DSM-IV criteria for alcohol dependence	Blood	Quantitative real-time PCR
Weischer et al., 2014 [49]	Prospective	4576 participants from the Copenhagen City Heart Study	38–68	43%	Alcohol consumption obtained from self-reported questionnaire	Blood	Quantitative real-time PCR
Yamaki et al., 2018 [50]	Cross-sectional	134 alcoholic patients (48 with upper aerodigestive tract cancer and 86 age-matched controls) and 121 non-alcoholic controls	58.7%	100%	Alcohol consumption obtained from the Kurihama Alcoholism Screening Test	Blood	Southern blot analysis of terminal restriction fragment lengths

**Table 2:** Findings from studies included in the systematic review.

Study	Main Results	Additional Findings
Aida et al., 2011 [37]	NTCR of basal cells was significantly larger in controls than in alcoholic patients	Basal cells had larger NTCR than parabasal cells
Aida et al., 2019 [38]	No difference in NTCR between non-drinkers and drinkers	No difference in NTCR between active or inactive ALDH2 genotypes
Dixit et al., 2019 [39]	At baseline and after 5 years of follow-up, TL was not different between alcohol consumers and alcohol abstainers. Weekly alcohol consumption did not correlate with TL	In Heart and Soul Study, binge drinking was associated with shorter TL. In Cardiovascular Health Study, no association between alcohol type and TL
Latifovic et al., 2015 [40]	No association between alcohol consumption and relative TL	Smoking status was associated with relative TL
Liu et al., 2013 [41]	No association between alcohol intake and relative TL	No relationships of folate, choline, methionine, riboflavin, vitamin B6, vitamin B12, and polymorphisms involved in one-carbon metabolism with relative TL
Martins de Carvalho et al., 2019 [42]	Alcohol use disorder was associated with lower relative TL. However, drinking behaviors were not associated with relative TL	A significant interaction between age and alcohol use disorder on relative telomere length was evident
Needham et al., 2013 [43]	No association between alcohol use and relative TL	The association between educational level and TL was partially mediated by smoking and body mass index but not by drinking or sedentary behavior
Pavanello et al., 2011 [44]	Relative TL was lower in alcohol abusers than in controls. The number of drinks per year was associated with relative TL in the overall population and among alcohol abusers	Polymorphisms in ADH1C and ALDH2 genes were not associated with TL
Révész et al., 2016 [45]	At the baseline, heavy drinking was associated with shorter TL if compared with moderate drinking	The association was not significant after adjusting for other predictors
Shin and Baik, 2016 [46]	No association between alcohol consumption and relative TL	An inverse association was found for heavy drinking among participants with mutant alleles of rs2074356 of ALDH2 gene
Strandberg et al., 2012 [47]	Age-adjusted TL was inversely associated with alcohol consumption at the baseline but not at the last follow-up	The association remained significant after adjusting for smoking, body mass index, cholesterol, perceived fitness
Tannous et al., 2019 [48]	Relative TL was lower in patients with alcohol disorder than in controls, but this difference was not statistically significant	NR
Weischer et al., 2014 [49]	No association between alcohol intake and TL	TL was associated with age, smoking status, body mass index, and physical inactivity
Yamaki et al., 2018 [50]	TL was shorter in patients with alcoholic disorders than controls	No association with cancer diagnosis, ADH1B and ALDH2 polymorphisms

Abbreviations: NTCR, Normalized telomere-to-centromere ratio; TL, Telomere length; NR, Not relevant.

**Table 3:** Comparison of characteristics between drinking and non-drinking women.

Characteristics	Drinkers (*n* = 5)	Non-Drinkers (*n* = 10)	*p*-Value
Age (years) ^a^	38.1 (4.2)	37.9 (3.9)	0.934
Gestational age at sampling (weeks) ^a^	16.1 (2.2)	16.2 (2.3)	0.937
Prepregnancy BMI (kg/m^2^) ^a^	24.2 (3.8)	24.0 (3.9)	0.926
Gestational age at delivery (weeks) ^a^	38.9 (2.1)	39.1 (2.0)	0.860
Fetal sex (male/female)	3/2	6/4	1.000

^a^ Results are reported as mean (standard deviations). Abbreviation: BMI, Body mass index.

## Data Availability

The data presented in this study are available on request from the corresponding author.

## Supplementary Materials

The following are available online at https://www.mdpi.com/article/10.3390/ijerph18095038/s1, Table S1: PRISMA Checklist.
